# Gambling Before and During the COVID-19 Pandemic Among European Regular Sports Bettors: An Empirical Study Using Behavioral Tracking Data

**DOI:** 10.1007/s11469-020-00327-8

**Published:** 2020-05-29

**Authors:** Michael Auer, Doris Malischnig, Mark D. Griffiths

**Affiliations:** 1 neccton GmbH, Davidgasse 5, 7052 Muellendorf, Austria; 2 Office of Addiction and Drug Policy of Vienna, Modecenterstrasse 14, Block B, 1030 Vienna, Austria; 3 International Gaming Research Unit, Psychology Department, Nottingham Trent University, 50 Shakespeare Street, Nottingham, NG1 4FQ UK

**Keywords:** Gambling, Online gambling, Online sports betting, COVID-19 pandemic, Online casino gambling

## Abstract

The novel coronavirus-2019 (COVID-19) pandemic has had major impacts on most societies worldwide including the cancelation and postponement of sports events. This has had a major impact on the sports betting industry. The present study is first to investigate the behavior of a sample of online sports bettors before and after COVID-19 measures were put in place by European governments. The authors were given access to the player data by a large European online gambling operator comprising players from Sweden, Germany, Finland, and Norway. The behavioral change of the sports bettors before March 7 and after March 7 (2020) was computed. All sports bettors who placed at least one wager in at least 5 calendar weeks out of the 10 possible calendar weeks between January 1 and March 7 (*n* = 5396) were included in the analysis. Results showed statistically significant reductions among sports bettors wagering in online casinos. This indicates that there was no conversion of money spent from sports betting to online casino games, at least for this particular online gambling operator. The findings suggest that there was a significant decrease in the amount of money wagered by sports bettors during the COVID-19 pandemic (compared with before it) and that sports bettors did not switch to playing more online casino games and that there was also a significant reduction in playing online casino games among sports bettors.

The World Health Organization (2020) declared the outbreak of the novel coronavirus-2019 (COVID-19) a pandemic on March 11 (2020) (Cucinotta and Vanelli 2020). The COVID-19 pandemic has had major impacts on most societies worldwide due to the way governments have implemented policies to inhibit the spread of the virus (e.g., quarantining, spatial distancing, national lockdowns, banning of events where large numbers of individuals congregate, etc.) (Pakpour and Griffiths 2020). One of the consequences of these safety measures has been the postponement or cancelation of almost all professional sporting events globally since early to middle March 2020 onwards (e.g., the cancelation of football (soccer) leagues in countries such as Belgium and France, the postponement of football leagues in countries like the UK and Germany, postponement of the Tokyo 2020 Olympics in Japan, cancelation of major tennis events like Wimbledon in the UK).

The cancelation and postponement of sports events in Europe (and elsewhere) has consequently had a major impact on the sports betting industry. Regulators and business experts have speculated about how players are reacting towards the fact they can no longer bet on sports games. Some regulators, policymakers, and treatment organizations have speculated in the mass media that individuals will gamble more online because of being in lockdown and spending so much of their daily lives at home and indoors (Davis 2020). The same groups of individuals have also wondered whether sports bettors (who are unable to gamble on sports events) might switch to gambling on other types of activity (e.g., online casino games) as an alternative. Utilizing behavioral tracking data, the present study is first to investigate the behavior of a sample of online sports bettors before and after COVID-19 measures were put in place by European governments.

Behavioral tracking data is increasingly being used for research purposes in the gambling studies field (Griffiths 2020). The use of account-based data compared to other methodologies (such as self-report surveys and experiments) has many advantages including objective data (as opposed to self-report data which is subject to many common methods biases such as social desirability and memory recall) and the fact that the sample sizes are usually much larger in their thousands or tens of thousands (e.g., Auer and Griffiths 2014, 2015, 2016, 2017, 2020; Braverman et al. 2013; Braverman and Shaffer 2012; Broda et al. 2008; Dragicevic et al. 2015; LaBrie et al. 2008, LaPlante et al. 2009; Leino et al. 2015, 2017; Nelson et al. 2008; Sagoe et al. 2018; Xuan and Shaffer 2009).

## Method

The authors were given access to the player data by a large European online gambling operator. The players were from Sweden, Germany, Finland, and Norway, and the authors carried out secondary analysis. The behavioral change of the sports bettors before March 7 and after March 7 (2020) was computed. In order to select regular sports bettors, the authors selected all sports bettors who placed at least one wager in at least 5 calendar weeks out of the 10 possible calendar weeks between January 1 (2020) and March 7 (2020). This resulted in a sample of 5396 sports bettors. The amount of money wagered on sports events and online casino games before and after March 7 (2020) was computed.

The product portfolio consists of sports betting and online casino games. Behavioral tracking data were available for a 4-month period from January 1 (2020) to April 30 (2020). The dataset allowed the authors to identify players who had wagered on sports or played online casino games. The authors computed the number of active sports betting players as well as the daily total bet across all sports betting players for each day between January 1 and April 30 (2020). The authors analyzed the data between January 1 and March 7 before the COVID-19 pandemic and data between March 7 and April 20 during the COVID-19 pandemic. The authors used the McNemar test to compare the changes in gambling participation before and after COVID-19. The McNemar test results in a chi-square test statistic and is applied for repeated measure comparisons with categorical data (Caronni and Sciumè 2017).

## Results

**Fig. 1:**
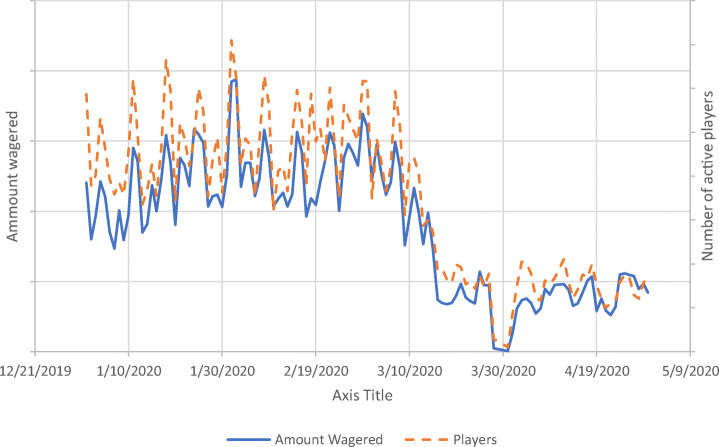
Time series of number of players (dashed line) and amount wagered (solid line) for each day between January 1, 2020, and April 30, 2020. Please note that actual player numbers and money spent are not provided for reasons of commercial sensitivity

Figure 1 displays the time series of the daily amount of money wagered and the daily number of active players. The time series shows that the highest amount of money wagered and the highest number of daily players from January 1 to March 10 (2020) were always Saturdays (i.e., the highest spikes in Fig. 1). The final spike which is in the range of the previous ones occurs on March 7, and after that, there was a rapid decline in the number of active players as well as in the daily total amount of money wagered. Social isolation measures due to the COVID-19 pandemic started in the various European countries around this time.

Because of this, the behavioral change of the sports bettors before March 7 and after March 7 (2020) was computed. In order to select regular sports bettors, the authors selected all players who placed at least one wager in at least 5 calendar weeks out of the 10 possible calendar weeks between January 1 (2020) and March 7 (2020). This resulted in a sample of 5396 sports bettors. The amount of money wagered on sports events and online casino games before and after March 7 (2020) was computed.

Table 1 shows the various sports and online casino wagering-specific statistics for sports bettors who wagered on sports in 5, 6, 7, 8, 9, or 10 different weeks between January 1 and March 7. The second row of Table 1 reports the percentage of players who were also active online casino players between January 1 and March 7. For example, three-quarters of the sports bettors who wagered on sports in 5 out of the 10 calendar weeks between January 1 and March 7 also played online casino games in the same time period (76%). The third row reports the percentage of sports bettors who also played online casino games between March 7 and April 30. For example, about two-thirds of sports bettors who wagered on sports in 5 out of the 10 calendar weeks between January 1 and March 7 also played online casino games between March 7 and April 30 (60%).

**Table 1:** Sports and online casino wagering-specific metrics for players who were active in sports betting for at least 5 calendar weeks between January 1, 2020, and March 7, 2020.

Number of distinct weeks with sports wagers	5	6	7	8	9	10
Played online casino games prior to March 7, 2020	76%	77%	77%	77%	78%	79%
Played online casino games after March 7, 2020	60%	62%	63%	68%	71%	76%
Ratio between wager per player in online casino games before and after March 7, 2020	54%	45%	56%	62%	71%	97%
*N*	1184	874	805	743	812	978

The changes between the two percentages (sports bettors playing online casino games before and after March 7) showed a statistically significant reduction in wagering among sports bettors in online casino games for all six groups. The chi-square values and *p* -values from the respective McNemar tests for the six groups are as follows: sports wagers during 5 separate calendar weeks, χ^2^ = 83.59, *p* < 0.001; 6 weeks, *χ*^2^ = 56.99, *p* < 0.001, 7 weeks, *χ*^2^ = 45.66, *p* < 0.001; 8 weeks, *χ*^2^ = 18.04, *p* < 0.001; 9 weeks, *χ*^2^ = 13.32, *p* < 0.001; and 10 weeks, *χ*^2^ = 5.05, *p* = 0.025. Table 1 also reports the ratio between the amount of money wagered per sports bettor in online casino games after March 7 compared with before March 7. The average wager per player in online casino games was 54% after March 7 compared with before March 7 for sports bettors who wagered in 5 out of the 10 calendar weeks before March 7. Sports bettors who wagered in every calendar week prior to March 7 produced 97% of the wagers in online casino games after March 7 compared with before March 7.

Table 2 reports the same metrics as Table 1. However, here sports bettors have been classified into ten equally sized groups according to the amount wagered on sports between January 1 and March 7. Group 1 comprises sports bettors with the lowest amount of money wagered during that time period, and group 10 comprises sports bettors with the highest amount of money wagered in that time period. Similar to Table 1, the percentage of sports bettors who played online casino games showed a statistically significant reduction in wagering among sports bettors in all ten groups after March 7, compared with the time period before March 7. The ten chi-square values and *p* values from the respective McNemar tests are as follows: group 1, *χ*^2^ = 12.34, *p* < 0.001; group 2, *χ*^2^ = 16.44, *p* < 0.001; group 3 *χ*^2^ = 22.48, *p* < 0.001; group 4 *χ*^2^ = 12.99 *p* = 0.0003; group 5 *χ*^2^ = 23.47, *p* < 0.0001; group 6 *χ*^2^ = 28.84, *p* < 0.0001; group 7 *χ*^2^ = 22.36, *p* < 0.0001; group 8 *χ*^2^ = 42.17, *p* < 0.0001; group 9 *χ*^2^ = 13.51, *p* = 0.0002; and group 10 *χ*^2^ = 11.82, *p* = 0.0005. Table 2 also reports the ratio between the amount of money wagered per sports bettor in online casino games after March 7 compared with before March 7 for each of the ten sports bettor intensity groups. The amount of money wagered per sports bettor in online casino games in group 1 was 60% after March 7 compared with before March 7.

**Table 2:** Sports and online casino wagering-specific metrics for players who were active in sports betting for at least 5 calendar weeks between January 1, 2020, and March 7, 2020, ranked by the amount wagered on sports before March 7, 2020

Equal sized groups according to wagering on sports before March 7, 2020	1	2	3	4	5	6	7	8	9	10
Played online casino games prior to March 7, 2020	85%	80%	78%	74%	73%	74%	75%	78%	77%	78%
Played online casino games after March 7, 2020	77%	71%	66%	66%	61%	61%	64%	60%	67%	68%
Ratio between wager per player on online casino games before and after March 7, 2020	60%	114%	87%	77%	60%	60%	38%	53%	57%	79%
*N*	540	539	540	539	546	534	538	540	539	540

## Discussion

The present study investigated the online casino gambling behavior of a European online gambling operator’s sports bettors before and during the COVID-19 pandemic. Results clearly showed that the daily active number of sports bettors and amount wagered on sports significantly decreased at around March 10 (see Fig. 1). After this date, sports betting activity was a much smaller proportion of what it was before. Tables 1 and 2 show that a large proportion of the sample of sports bettors also played online casino games before COVID-19 between January 1 and March 7. This means that most sports bettors (> 70%) were also online casino players during this period.

The authors first classified sports bettors according to the number of distinct weeks with at least one wager. Players had to have wagered in at least 5 of the 10 possible calendar weeks before March 7 (2020) to be defined as a regular sports bettor. In all of the groups, the percentage of sports bettors who played online casino games was significantly lower after March 7 compared with before March 7. This means that not only did players wager less on sports (most events had been canceled by March 7), but they also wagered less on online casino games. This indicates that there was no conversion of money spent from sports betting to online casino, at least for this particular online gambling operator.

The lowest difference between percentage of online casino players before and after March 7 occur red among the two groups of sports bettors who wagered for 9 out of 10 calendar weeks and all 10 calendar weeks. The percentage of sports bettors who played online casino games before March 7 was 78% and after March 7 was 71% for those who wagered in 9 calendar weeks. The percentage of sports bettors who played online casino games before March 7 was 79% and after March 7 was 76% for the ones who wagered in 10 calendar weeks. Both differences were statistically significant, but of lower significance than for sports bettors who wagered in only 5 calendar weeks. The respective percentages for those who only wagered in 5 out of 10 calendar weeks were 76% before March 7 and 60% after March 7.

Although online casino gambling did not become more frequent, it appears that more frequent sports bettors also maintained their online casino gambling, whereas less frequent sports bettors were more likely to stop gambling altogether. This conclusion does not apply to sports betting gambling intensity. Table 2 shows ten groups of sports bettors according to the amount bet between January 1 and March 7. The change in the percentage of online casino gambling is similar across all intensity groups. More intense sports bettors did not appear to play online casino games more or less often when sports betting was not available during the COVID-19 pandemic.

The ratio of the amount of money wagered on online casino games per player before and after March 7 was also computed. Table 1 clearly shows that sports bettors who wagered over more weeks did not decrease their average amount wagered as much as sports bettors who wagered over less weeks. Most frequent sports bettors’ (wagered in 10 calendar weeks) average casino wager after March 7 was 97% of their average casino wager before March 7. On the other hand, the same percentage was 57% for sports bettors who only placed a bet during 5 calendar weeks. This means that frequent sports bettors’ average amount of money wagered at the online casino games stayed the same. This was not observed with respect to monetary intensity in sports betting. The 10% of sports bettors with the highest amount wagered between January 1 and March 7 did not increase or decrease their amount wagered more than any other intensity group. There was no clear association between the amount of money wagered on sports betting and change in average amount wagered in online casino games.

The present study includes some limitations that should be taken into account when interpreting the findings. Firstly, the results of present study were derived from data from a single European online gambling operator’s player base comprising four European nationalities (Sweden, Norway, Finland, and Germany). This online gambling operator’s player base is from specific countries, and players from other countries might have gambled differently during the COVID-19 lockdown and the lack of sports events to wager upon. Secondly, the totality of gambling behavior among the participants is unknown because the dataset only includes the gambling behavior of the participants at this specific online gambling operator. Thirdly, there is a possibility that some accounts may have been used by more than one gambler (e.g., a husband and wife or a father and son) although the number of occurrences is likely to be low. Fourthly, the study’s dataset is derived from the time period from January to April 2020 (inclusive). Because the lockdown as a consequence of the COVID-19 pandemic was still active in many countries as of April 30 (and most sports events were still postponed or canceled), sports bettors may have started to play casino games more intensely after April 30.

Findings from the present study suggest that there is no relationship between the lack of sports betting events for gamblers to wager upon and increased frequency and intensity of online casino gambling. Overall, the frequency of wagering upon online casino games by online sports bettors before COVID-19-related lockdown significantly decreased during the COVID-19 pandemic period. However, less frequent (but regular) online sports bettors significantly decreased the amount of money wagered upon online casino games more than more frequent (regular) online sports bettors. Frequent online sports bettors wagering upon online casino games stayed approximately the same before and during the COVID-19 pandemic.

These findings suggest that the speculations that individuals may spend more time and money gambling online as a consequence of being confined in their house for long periods of time appear unfounded. The decrease in overall gambling might be due to a number of factors including individuals having less money to gamble because their occupational earning potential has been lower during the pandemic, individuals not wanting to gamble in front of their family members, or individuals spending more time on other activities such as spending “quality time” with their families or finally having the time to do bigger jobs around the house and garden (e.g., decorating and home and garden improvements). The present study is the first to empirically investigate whether wagering increases or decreases among gamblers (in this case, sports bettors specifically) during a period when many individuals are housebound during the COVID-19 pandemic-related lockdowns.
